# The relationship between cortisol, C-reactive protein and hypertension in African and Causcasian women: the POWIRS study

**DOI:** 10.5830/CVJA-2011-035

**Published:** 2012-03

**Authors:** Claire M Tolmay, Leone Malan, Johannes M Van Rooyen

**Affiliations:** Hypertension in Africa Research Team (HART), North-West University, Potchefstroom, South Africa; Hypertension in Africa Research Team (HART), North-West University, Potchefstroom, South Africa; Hypertension in Africa Research Team (HART), North-West University, Potchefstroom, South Africa

**Keywords:** cortisol, C-reactive protein, hypertension, ethnic

## Abstract

Research on the roles that C-reactive protein (CRP) and other risk factors such as cortisol and obesity play in the diagnosis of cardiovascular disease (CVD) in African and Caucasian women has become increasingly imperative when one considers the prevalence of hypertension in these groups. CRP and cortisol have been associated with an increased prevalence of hypertension and obesity. Cortisol has also been linked with both hypertension and the hypothalamic–pituitary–adrenal (HPA) response. African women have previously presented with an increased vascular reactivity. Conversely, Caucasian women have displayed an increased central cardiac reactivity. We included African (*n* = 102) and Caucasian (*n* = 115) women in the study, matched for age and body mass index. Elevated CRP levels were observed in African women compared to Caucasian women. A trend of hypocortisolism was exhibited in both hypertensive ethnic groups. Systolic blood pressure (SBP) and a vascular marker, arterial compliance (Cw), predicted hypertension in African women. Conversely, in Caucasian women, only SBP predicted hypertension. These results suggest the apparently diverse roles that dysregulation by the HPA axis, in conjunction with the respective cardiac and vascular responses in both Caucasian and African women, can play in future cardiovascular risk for these groups.

Several studies in southern Africa have explored the relationship between C-reactive protein (CRP), cortisol and the prevalence of hypertension, and cardiovascular, anthropometric and other risk factors in the development of cardiovascular disease (CVD).^1^-^7^ These factors include the influence of urbanisation in different ethnic groups.^2^-^7^ Urbanisation has been shown to play a significant role in increasing cardiovascular reactivity in Africans when compared to other ethnic groups.^2^,^3^,^5^ These studies have also demonstrated a correspondingly higher prevalence of hypertension in this group compared to Caucasians, Indians and those of mixed origin.^2^,^3^,^5^

African women have previously been shown to have significantly higher high-sensitivity C-reactive protein (hs-CRP) and blood pressure (BP) levels compared to their Caucasian counterparts. However, no cardiovascular parameters could explain the variation in levels of this inflammatory marker.^6^ Caucasian women, however, did show strong significant correlations between CRP, Windkessel compliance (Cw) and total peripheral resistance (TPR).^6^ Nevertheless these correlations became non-significant and weak after adjustments were made for age, body mass index (BMI) and waist circumference (WC).^6^

Recently, Hamer and Malan^6^ have revealed that urban Africans had higher BP and hypertension (HT) rates in conjunction with higher arterial resistance and lower cardiac output compared to their rural counterparts. The exact mechanism of the increased prevalence of hypertension in this ethnic group is still unknown but great strides have been made in the determination of other possible risk factors such as psychosocial stress and coping responses.^1^,^6^,^7^ Hypocortisolism, in conjunction with urbanisation, has been proposed as a possible contributing factor to the increased incidence of hypertension in African women,^4^ however further studies need be conducted in order to assess what role this observation plays in association with other risk factors for CVD in this ethnic group.

Several studies have shown that acute or chronic stress may lead to dysregulation of the hypothalamic–pituitary–adrenal (HPA) axis, which in turn is mediated by certain inflammatory markers such as CRP and other stress hormones, including cortisol.^8^-^11^ Free fatty acids, which are released from increased visceral or abdominal adipose tissue, also play a role in heightening levels of CRP.^11^-^14^ Elevated levels of cortisol are also linked to increased adiposity and subsequently increased inflammation, which consequently leads to increased risk for cardiovascular disease.^10^,^11^ Moreover, this link is further supported by the decrease in inflammatory mediators in individuals where weight loss was demonstrated.^15^

It is important to note, however, that although several of these studies have explored in detail the relationships between cortisol, CRP and vascular responses, and the prevalence of hypertension, and that although guidelines have been set for other ethnic groups,^15^ the investigation of African and Caucasian women from South Africa has been limited. It is therefore imperative that the relationship between cortisol, CRP and vascular responses, and the prevalence of hypertension be explored further in the ethnic groups of South Africa in order to fully substantiate and assess possible risk for CVD.

## Methods

The POWIRS (Profiles of Obese Women with Insulin Resistance Syndrome) study comprised two cross-sectional studies, of which the first was performed on 102 urban African women. This study was then repeated on a group of 115 Caucasian women. The two groups were matched on the basis of age and BMI. Attempts were made to choose African women of a higher socio-economic status (living area, household composition, income and educational level).^16^ These women were recruited from among the employees of a government institution. The dietician in this institution recruited the subjects while taking the initial study design into account.

An attempt was made to include Caucasian women from the same institution, but since only a very small percentage of women employed by the institution were Caucasian, we had to include women from other institutions as well. The research nurse who was involved in the second phase of the study recruited the Caucasian women, based on the characteristics of the African women, namely, for each urban African woman who was, for example, 30 years of age, with a BMI of 25 kg/m^2^ and employed, a similar Caucasian woman with these characteristics was sought.

All participants lived in the Potchefstroom district, South Africa. Exclusion criteria were pregnancy, lactation, diabetes mellitus or an oral temperature above 37°C. CRP readings were only available for 101 African subjects.

Following the introduction of the subjects to the experimental set up and an explanation of the procedures used, they each signed an informed consent form. The Ethics Committee of the North-West University approved the study, and all procedures followed were in accordance with institutional guidelines. Each subject received a participant sheet that guided her through the different research stations, and was signed at each station.

All anthropometric measurements (except height and weight measurements) were taken during the course of the evening, after which all participants received an identical supper at 20:00, which excluded alcohol or caffeine. All subjects were asleep before 23:00 and fasted overnight. At 6:00 the following morning, weight, height and blood pressure measurements were obtained, followed by blood sampling at 08:00. They then received breakfast and personal information sheets regarding their own blood pressure, blood glucose levels, etc, and indicating where further testing and/or treatment were necessary. All subjects were given a small financial compensation and were transported back to their places of work.

All data were collected from the African subjects during March and April 2003, and the samples were assayed during August 2003. Data collection on the Caucasian subjects was done during August 2004 and the assays were completed during October 2004.

Height (stature), weight and waist circumference of the subjects were measured by a qualified anthropometrist with calibrated instruments using standard methods (Precision Health scale, A & D Company, Japan; Invicta Stadiometer, IP 1465, UK; Holtain unstretchable metal tape; John Bull callipers). All measurements were standardised and taken in triplicate.

A seven-minute resting continuous measurement of cardiovascular parameters was taken while in the supine position using the Finometer device (Finapres Medical Systems®, Amsterdam, Netherlands). The Beatscope® version 1.1 software further calculated an integrated age-dependent aortic flow curve from the surface area beneath the pressure/volume curve, determining each subject’s heart rate (HR), systolic (SBP) and diastolic (DBP) blood pressure, stroke volume (SV), cardiac output (CO), total peripheral resistance (TPR) and arterial compliance (Cw) of the small and large arteries (Finapres Medical Systems®, Amsterdam, Netherlands). Duplicate blood pressure readings were taken using a single-headed stethoscope and a mercury sphygmomanometer (model ALPK2) both before and after the Finometer measurements. The first and fifth Korotkoff phases were recorded as the SBP and DBP, respectively.

hs-CRP levels were determined using blood serum samples that were analysed with a high-sensitivity C-reactive protein kit from Immage® Immunochemistry Systems (Cat no. 474630, Beckham Coulter, Inc). Serum cortisol was measured with ^125^I RIA Coat-a-count kit (Diagnostic Products Corporation, Cat no. TKC01). The intra- and inter-assay coefficients of variation for cortisol were, respectively, 7.7 and 9.8%.

## Statistical analyses

Data were analysed using the software computer package STATISTICA 9.0. Departure from normality was evaluated through Shapiro-Wilk’s analyses and hs-CRP and cortisol were log transformed. All data was corrected for age, smoking and alcohol consumption. Independent *t*-tests were performed to compare the two ethnic groups in terms of age, anthropometric, cardiovascular and biochemical variables. Partial correlations were performed to determine the correlations of both CRP and cortisol with various anthropometric and cardiovascular variables, while adjusting for age, smoking and alcohol consumption.

The subjects of each ethnic group were stratified into normotensive (NT) and hypertensive (HT) groups according to WHO recommendations to determine their hypertensive status.^17^ Significant differences in each ethnic group were determined by ANCOVA analyses for both cardiovascular and anthropometric variables, while adjusting for age, smoking and alcohol consumption. Stepwise forward regression analyses were used in each ethnic group to predict the relationship between cardiovascular and anthropometric variables and hypertension.

## Results

Table 1 represents the cardiovascular and anthropometric characteristics of the African and Caucasian women. Waist circumference was the only anthropometric variable that exhibited significantly higher values in Caucasian women compared to African women. Conversely, SBP and the vascular markers TPR and DBP were increased in African women compared to their Caucasian counterparts. In the Caucasian women the cardiac markers CO and SV, and cortisol values were increased compared to African women. Both ethnic groups exhibited elevated CRP levels (> 3.0 mg/l).

**Table 1:** Descriptive Statistics Of Cardiovascular And Anthropometric Variables Between African And Caucasian Women

	*African women mean (95 % CI) n = 102*	*Caucasian women mean (95% CI) n = 115*
Age	31.12 (29.43; 32.81)	30.98 (29.17; 32.79)
WC (cm)	81.62 (79.02; 84.22)^a^	85.98 (83.18; 88.76)^a^
BMI (kg/m^2^)	27.98 (26.74; 29.23)	28.51 (27.16; 29.85)
SBP (mmHg)	129.82 (125.97; 133.66)^b^	125.24 (123.03; 127.45)^b^
DBP (mmHg)	77.68 (75.58; 79.78)^c^	72.30 (70.61; 73.99)^c^
CO (l/min)	5.72 (5.49; 5.95)^d^	7.09 (6.73; 7.45)^d^
SV (ml)	84.67 (81.84; 87.51)^e^	97.94 (93.14; 102.74)^e^
TPR (mmHg/s/ml)	1.10 (1.04; 1.15)^f^	0.84 (0.80; 0.88)^f^
Cw (ml/mmHg)	1.85 (1.79; 1.91)^g^	2.29 (2.21; 2.36)^g^
hs-CRP (mg/l)	5.54 (3.19; 7.88)	3.29 (2.57; 4.01)
Cortisol (nmol/ml)	455.74 (402.37; 509.11)^h^	604.90 (545.23; 664.56)^h^

CI, confidence interval; WC, waist circumference; BMI, body mass index; SBP, systolic blood pressure; DBP, diastolic blood pressure; CO, cardiac output; SV, stroke volume; TPR, total peripheral resistance; Cw, Windkessel compliance; hs-CRP, high-sensitivity C-reactive protein. All data were adjusted for age, smoking and alcohol consumption and tested at a 95% confidence interval. Significant differences (*p* ≤ 0.05) between variables in the groups are indicated with the same superscript letters.

Table 2 represents the overall mean characteristics of the normotensive/hypertensive Caucasian and African women subjects after adjusting for age, smoking and alcohol usage. There was an overall trend of a significant increase in mean values of WC and BMI in the African women once the hypertensive range (≥ 140/90 mmHg) was approached.

**Table 2:** Cardiovascular And Anthropometric Variables Between Normotensive And Hypertensive African And Caucasian Women

	*African women*		*Caucasian women*	
	*Normotensive (NT) n = 81*	*Hypertensive (HT) n = 21*	*Normotensive (NT) n = 100*	*Hypertensive (HT) n = 12*
Age	30.02 (28.28; 31.77)^a^	36 (31.49; 40.51)^a^	30.76 (28.94; 32.59)	35.58 (29.15; 42.02)
BMI (kg/m^2^)	27.31 (25.96; 28.65)^b^	30.59 (27.51; 33.67)^b^	27.94 (26.54; 29.34)	31.79 (26.54; 37.05)
WC (cm)	80.01 (77.17; 82.86)^c^	87.81 (81.83; 93.79)^c^	84.54 (81.65; 87.42)^h^	94.60 (84.37; 104.83)^h^
SBP (mmHg)	122.93 (120.90; 124.96)^d^	156.37 (144.58; 168.15)^d^	122.05 (120.43; 123.67)^i^	146.91 (138.81; 155.00)^i^
DBP (mmHg)	74.34 (72.50; 76.18)^e^	90.58 (86.53; 94.64)^e^	70.89 (69.27; 72.51)^j^	82.12 (75.12; 89.13)^j^
hs-CRP (mg/l)	5.55 (2.64; 8.45)	5.50 (3.04; 7.95)	3.27 (2.45; 4.10)	3.50 (2.05; 4.95)
Cortisol (nmol/ml)	462.24 (397.84; 526.63)	430.68 (348.22; 513.15)	610.29 (545.70; 674.88)	534.87 (318.67; 751.07)

CI, confidence interval; hs-CRP, high-sensitivity C-reactive protein; WC, waist circumference; BMI, body mass index; SBP, systolic blood pressure; DBP, diastolic blood pressure. All data were adjusted for age, smoking and alcohol consumption and tested at a 95% confidence interval. Significant differences (p ≤ 0.05) between variables in the groups are indicated with the same superscript letters.

**Fig. 1:**
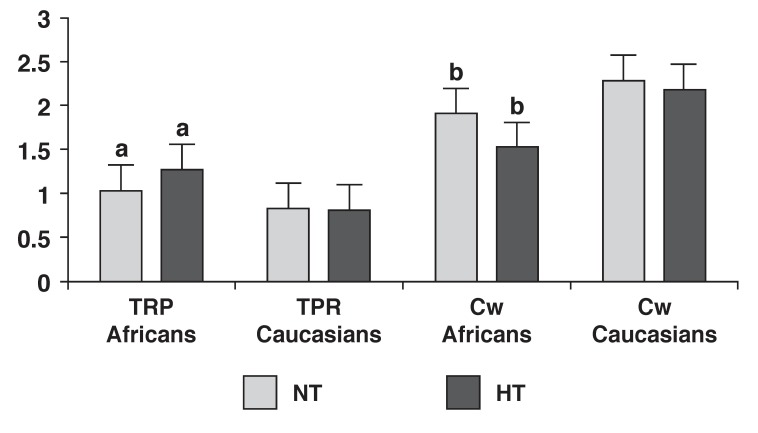
Resting mean (± SD) vascular responses of African and Caucasian women between normotensive (BP ≤ 140/90 mmHg) and hypertensive (BP ≥ 140/90 mmHg) groups. SD, standard deviation; BP, blood pressure; TPR, total peripheral resistance (mmHg/s/ml); Cw, Windkessel compliance (ml/mmHg); NT, normotensive; HT, hypertensive. Values are adjusted for age, smoking and alcohol consumption and tested at a 95% confidence interval. Bars with the same superscript letter differ significantly.

**Fig. 2:**
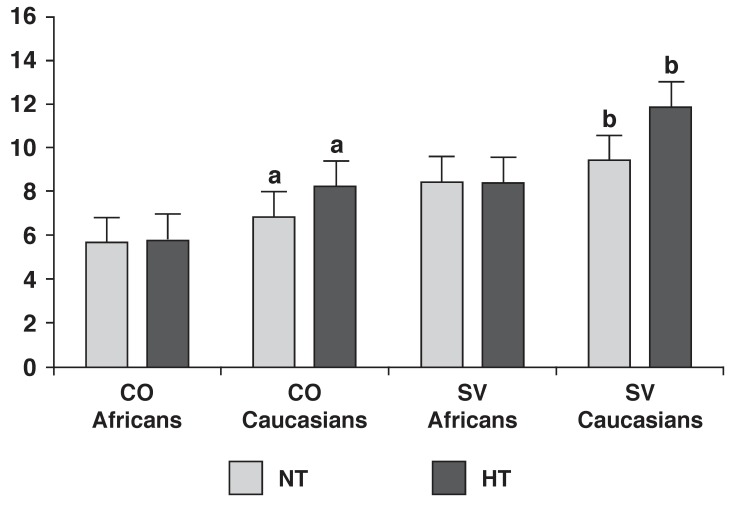
Resting mean (± SD) cardiac responses of African and Caucasian women between normotensive (BP ≤ 140/90 mmHg) and hypertensive (BP ≥ 140/90 mmHg) groups. SD, standard deviation; BP, blood pressure; CO, cardiac output (l/min); SV, stroke volume (l); NT, normotensive; HT, hypertensive. Values are corrected for age, smoking and alcohol consumption and tested at a 95% confidence interval. Bars with the same superscript letter differ significantly.

In Fig. 1, increased vascular responses (increased TPR and decreased Cw) were demonstrated in African women compared to Caucasian women. The central cardiac pattern (increased SV and CO) seen in Fig. 2 showed a propensity to significantly increase (*p* ≤ 0.05) from normotensive to hypertensive values in Caucasian women.

In Table 3, hypertensive African women exhibited significant positive associations between log CRP and both WC and BMI. Caucasian hypertensive women showed significant correlations of log CRP with both SV and Cw (Table 3). Conversely, hypertensive African women showed highly significant correlations of log CRP with all cardiovascular variables (excluding DBP) (Table 3).

**Table 3:** Significant Associations Between Cardiovascular And Anthropometric Variables And High-Sensitivity Log Crp In Normotensive And Hypertensive African And Caucasian Women

	*African women*				*Caucasian women*			
	*Log CRP: normotensive*		*Log CRP: hypertensive*		*Log CRP: normotensive*		*Log CRP: hypertensive*	
	r	p	r	p	r	p	r	p
WC (cm)	0.46	0.00	0.69	0.03	0.39	0.01	0.53	0.45
BMI (kg/m^2^)	0.37	0.02	0.80	0.00	0.43	0.00	0.52	0.48
CO (l/min)	0.28	0.18	0.66	0.04	0.29	0.15	0.52	0.47
SV (ml)	0.19	0.56	0.73	0.01	0.21	0.47	0.84	0.01
TPR (mmHg/s/ml)	0.28	0.17	0.74	0.01	0.31	0.09	0.59	0.33
Cw (ml/mmHg)	0.24	0.34	0.85	0.00	0.18	0.63	0.83	0.01
SBP (mmHg)	0.31	0.10	0.79	0.00	0.81	0.97	0.53	0.47
DBP (mmHg)	0.16	0.73	0.64	0.06	0.14	0.84	0.62	0.26

CRP, C-reactive protein; WC, waist circumference; BMI, body mass index; CO, cardiac output; SV, stroke volume; TPR, total peripheral resistance; Cw, Windkessel compliance; SBP, systolic blood pressure; DBP, diastolic blood pressure; SD, standard deviation. All data were adjusted for age, smoking and alcohol consumption and tested at a 95% confidence interval. Significant differences (*p* < 0.05) are highlighted in bold. Significant differences (*p* ≤ 0.05) between variables in the groups are indicated with the same superscript letters.

In Table 4, the hypertensive Caucasian women showed positive associations between log cortisol and the vascular markers Cw and DBP. On the other hand, hypertensive African women only exhibited positive associations between log cortisol and TPR and SBP.

**Table 4:** Significant Associations Between Cardiovascular And Anthropometric Variables And Log Cortisol In Normotensive And Hypertensive African And Caucasian Women

	*African women*				*Caucasian women*			
	*Log cortisol: normotensive*		*Log cortisol: hypertensive*		*Log cortisol: normotensive*		*Log cortisol: hypertensive*	
	r	p	r	p	r	p	r	p
WC (cm)	0.46	0.00	0.23	0.93	0.38	0.02	0.53	0.46
BMI (kg/m^2^)	0.35	0.04	0.28	0.84	0.41	0.00	0.52	0.48
TPR (mmHg/s/ml)	0.28	0.18	0.66	0.048	0.32	0.09	0.59	0.32
Cw (ml/mmHg)	0.23	0.36	0.82	0.00	0.18	0.66	0.81	0.02
SBP (mmHg)	0.24	0.36	0.73	0.01	0.16	0.72	0.64	0.22
DBP (mmHg)	0.14	0.84	0.62	0.09	0.18	0.65	0.80	0.03

CRP, C-reactive protein; WC, waist circumference; BMI, body mass index; CO, cardiac output; SV, stroke volume; TPR, total peripheral resistance; Cw, Windkessel compliance; SBP, systolic blood pressure; DBP, diastolic blood pressure; SD, standard deviation. All data were adjusted for age, smoking and alcohol consumption and tested at a 95% confidence interval. Significant differences (*p* < 0.05) are highlighted in bold. Significant differences (*p* ≤ 0.05) between variables in the groups are indicated with the same superscript letters.

Stepwise forward regression analyses (Table 5) revealed that the cardiac (SBP and SV) and vascular markers (Cw) predicted hypertension in African women. In Caucasian women, only SBP predicted hypertension.

**Table 5:** Stepwise Forward Regression Analyses Of Cardiovascular And Anthropometric Variables Predicting Hypertension In Caucasian And Black Women

	*Caucasian women*			*African women*		
	*Adjusted R^2^*	β* (± SE)*	p-*value*	*Adjusted R^2^*	β* (± SE)*	p-*value*
SBP	0.81	0.69 (0.11)	0.00	0.99	1.58 (0.13)	0.00
SV	0.84	0.59 (0.12)	0.39	0.94	0.15 (0.11)	0.02
Cw	0.79	–0.96 (0.01)	0.14	0.97	–0.18 (0.10)	0.03

β denotes standardised regression coefficient and *F* to enter model, 2.5. Analyses adjusted for age, BMI, WC, CO, TPR, log CRP and log cortisol.

## Discussion

The aim of this study was to investigate the contribution of CRP, cortisol and hypertension to the increased likelihood of cardiovascular disease in both African and Caucasian women from South Africa. This study is relevant in that it describes the relationship between various cardiovascular risk markers and hypertension in two ethnic groups, which, as far as we have ascertained, have been neglected.

Hypertensive Caucasian women exhibited an increased central cardiac activity, as demonstrated by the increased CO (Table 2, Fig. 2). There was also a significant difference between normotensive and hypertensive SBP and DBP values, respectively, for Caucasian women (Table 2). These observations, in conjunction with the decreased cortisol levels in the hypertensive group, suggest increased sympathetic reactivity with an accompanying hypocortisolism. This corresponds with recent studies,^4^,^10^,^18^ where it was found that hypocortisolism has in some cases been related to hypertension.

Hypertensive African women, conversely, showed increased vascular reactivity (Table 2, Fig. 1). Cw significantly decreased in this group, with a corresponding significant increase in TPR. Hypocortisolism was also exhibited in the hypertensive African women (Table 2). This possibly suggests an over-stimulated HPA axis, leading to hypertension in association with hypocortisolism.^4^,^10^,^18^

Urbanisation has previously been shown to be a psychosocial stressor that contributes to increased cardiovascular reactivity and hence, hypertensiveness.^1^-^6^ Since the African women in this study were urbanised, one could hypothesise that this could be a possible explanation for these results.^16^ Specifically, the increased vascular reactivity in the hypertensive African women corresponds to previous studies that found an increased relative risk for hypertension in both Africans and African-Americans compared to other ethnic groups, due to increased measurements of cardiovascular reactivity when a specific stressor was applied.^2^,^3^,^5^,^19^-^23^

CRP was significantly correlated with WC and BMI in the hypertensive African women. However, this observation did not apply to the Caucasian women (Table 3). These observations agree with previous studies, where it was shown that CRP had its strongest correlations with BMI and WC,^7^,^24^-^26^ and that these correlations were most noticeable among African-American and African women.^7^,^27^

Mean values of BMI and WC in hypertensive African women also significantly increased (Table 2). This increase in mean body fat indicators could possibly explain the high correlations with CRP when one considers the relationship between IL-6 and CRP concentrations. IL-6 is expressed in adipose tissue and this inflammatory marker in turn stimulates the production of CRP in the liver.^24^,^28^ In addition, IL-6 has been shown to increase with increasing adiposity in healthy men and women, with higher amounts being expressed in visceral fat depots compared to subcutaneous fat depots.^12^,^13^,^29^ This mechanism could possibly explain the observed higher correlation with WC compared to BMI in African women, as BMI represents overall adiposity whereas WC represents abdominal adiposity.

This could possibly be attributed to the observation that the normotensive Caucasian and African women had already exhibited elevated mean values of both BMI and WC (Table 2) before reaching hypertensive status. These individuals could already have been exhibiting reactivity of the HPA axis^9^-^11^,^29^-^32^ to the elevated adiposity, and therefore a significant correlation to both CRP and cortisol was seen. Since the duration of obesity was unknown, the loss of these correlations in the hypertensive groups could possibly be attributed to dysregulation of the HPA axis,^4^,^10^,^18^ which was further supported by the observed hypocortisolism (Table 2).

CRP was associated with all cardiovascular variables in the hypertensive African women. However, the highest correlations were exhibited with the peripheral vascular reactivity indicators, TPR and Cw (Table 3). Once again, these observations, in conjunction with the significant differences between the mean values of these indices (Table 2), suggest a complementary peripheral cardiovascular reactivity accompanying an increase in blood pressure in African women.

This, together with the significant associations with CRP in this group, is congruous with previous studies where CRP was reported to be associated with hypertensive blood pressure values, with greater associations occurring among women and black ethnic groups.^28^,^29^,^33^-^42^ In addition, increases in blood pressure in obese African-American women have previously been demonstrated, with a greater indexed peripheral resistance, taking into account body surface area and not BMI (compared to Caucasians).^21^,^43^ Moreover, CRP levels have previously corresponded to an increased likelihood of peripheral arterial disease in United States adults, independent of smoking, WC, BMI, hypertension and other confounders.^44^

It can therefore be deduced that in African women, the high correlations between CRP and the peripheral cardiovascular variables TPR and Cw suggest a greater risk for the development of hypertension and consequently the development of cardiovascular disease. In addition to these observations, the overall mean values of CRP were higher in Africans compared with Caucasians (Table 1). However, both ethnic groups demonstrated CRP levels that fell within the high-risk group for CVD (> 3.0 mg/l).^45^ Caucasian women displayed a significant correlation between CRP and both SV (*r* = 0.84; *p* = 0.01) and Cw (*r* = 0.83; *p* = 0.01) (Table 3).

Previous studies have found that increased CRP levels have been associated with decreased nitric oxide production.^37^,^45^ Inhibition of nitric oxide in turn leads to increased α-adrenergic effects, including increased vasoconstriction and sympathetic activity.^46^ We suggest that the inhibition of nitric oxide production could possibly explain the decrease in Cw and the significant increases in CO and SV values (Table 2), in conjunction with the high correlations of CRP with SV and Cw (Table 3). Therefore the increased CRP in Caucasian women could feasibly cause endothelial dysfunction, which in turn could lead to hypertension and subsequent cardiovascular dysfunction. Both Caucasian and African women therefore exhibited possible risk markers for future cardiovascular disease.

Cortisol correlated significantly with both Cw and TPR in the hypertensive African women, whereas in the Caucasian women there was a significant correlation with only Cw (Table 4). Decreased cortisol levels were also exhibited in both hypertensive ethnic groups (Table 2). This could be indicative of hypocortisolism, which has previously been displayed in hypertension, particularly in African women.^4^,^10^,^18^ Suggestions have been made that this could be attributed to dysregulation of the HPA axis, which in turn is mediated by certain inflammatory markers such as CRP.^15^ Therefore the high correlation between Cw and TPR in these ethnic groups could still be attributed to the initial effects of increased cortisol production by the HPA axis in response to stress. This in turn could further support the significant increase in the cardiac markers CO and SV in the hypertensive Caucasian women (Table 2), and the significant increase in means of TPR and Cw in the hypertensive African women (Table 2).

A possible mechanism for this could be the permissive effect of norepinephrine release by cortisol stimulation and subsequent β-adrenergic responses.^46^ In the hypertensive African women, SBP and the vascular marker Cw predicted hypertension (Table 5), suggesting possible dysregulation of the HPA axis in conjunction with norepinephrine release by cortisol stimulation in this group.^4^,^10^,^15^,^18^,^46^ Therefore in the hypertensive African women, dysregulation of the HPA axis is the most likely explanation. However, in the hypertensive Caucasian women, these observations were not reiterated.

A limitation of this study could be the duration of obesity in the participants, which could have influenced the habituation/adaptation of physiological resources. Furthermore, one needs to consider that hs-CRP is a non-specific marker for inflammation and although the participants in this study were apparently healthy, the higher hs-CRP levels could possibly be explained by other unknown inflammatory conditions.^45^ Additionally, the time difference in data collection could have influenced the results, although temperatures were similar for both collection periods (autumn and spring). The significance of this study could have been further substantiated if more data were obtained regarding duration of stay in an urban area. Additionally, the use of hormone replacement therapy or oral contraceptives should have been ascertained, as these could have affected the CRP values in this study.

## Conclusion

Both African and Caucasian women displayed possible dysregulation of the HPA axis, which could explain the hypertension in these groups. The roles of CRP and cortisol remain uncertain in these groups. However, in the Caucasian women, cortisol presented a higher likelihood of contribution towards hypertension than in the African women, whereas in the hypertensive African women, CRP seemed to play a larger role (Tables 3, 4). Future studies should include further examination of the role of dysregulation of the HPA axis in these groups and the possible mechanisms of action regarding the incidence of hypertension considering these effects.
